# Reliability of nurse-administered infant hearing screening using otoacoustic emissions

**DOI:** 10.4102/sajcd.v72i1.1092

**Published:** 2025-07-25

**Authors:** Mukovhe Phanguphangu, Andrew J. Ross

**Affiliations:** 1 Department of Family Medicine, College of Health Sciences, University of KwaZulu-Natal, Durban, South Africa

**Keywords:** congenital hearing loss, Infant hearing screening, nurse-administered screening, reliability studies, otoacoustic emissions screening, rural setting

## Abstract

**Background:**

In South Africa (SA), congenital hearing loss (HL) is identified at around 30 months of age, which is later than local standards of identification by 6 weeks, mainly because of limited access to infant and newborn hearing screening (INHS). Thus, there is a critical need to explore other models of providing early detection such as nurse-administered INHS.

**Objectives:**

This study aimed to determine the reliability of nurse-administered INHS.

**Method:**

This was a repeated-measures study where 50 infants scheduled to receive their 6-week immunisation were independently screened by two nurses and an audiologist using distortion product otoacoustic emissions (DPOAE). Data were analysed using Cohen’s kappa, using Stata v18 for Macintosh.

**Results:**

Thirteen (*n* = 13, 26%) infants failed DPOAE screening tests, of which four were male and nine (*n* = 9) were female. All participants who failed the screening were referred to the hospital for further evaluation and intervention as needed. Further analysis revealed an almost perfect agreement between audiologist- and nurse-administered screening (*k* = 0.81, *p* < 0.001).

**Conclusion:**

Findings from this study demonstrate that nurses can consistently screen and identify babies with congenital HL using DPOAE screening tests. Furthermore, these findings pave the way for incorporating nurse-administered DPOAE screening into immunisation programmes, with the potential to increase access to INHS and reduce the age of identification of congenital HL to acceptable standards. Large-scale research is recommended to explore the implementation of this nurse-administered INHS in other contexts.

**Contribution:**

This study contributes to the growing body of evidence on INHS in SA.

## Introduction

In South Africa (SA), more than 6100 babies are born with or acquire hearing loss (HL) in the first weeks of life annually (Swanepoel et al., 2009). This HL often goes undetected at birth and when infant and newborn hearing screening (INHS) is not performed, the HL can go unnoticed for months to years (Phanguphangu, 2017; Phanguphangu et al., 2024). Infant and newborn hearing screening can facilitate the early detection and intervention of this HL (Phanguphangu et al., 2024). However, most newborns do not receive INHS in SA because of systemic challenges such as the early discharge of newborns from hospitals before hearing screening can be conducted (Phanguphangu et al., 2025). This results in a late detection of congenital HL at an average of 30 months of age (Phanguphangu et al., 2025), which is long after the critical developmental periods for language acquisition have passed (The Joint Committee on Infant Hearing, 2019). Unfortunately, this late detection has devastating consequences on communication development as limited access to sound often inhibits speech, language and psychosocial development (Tomblin et al., 2015). These consequences are cumulative, leading to poor scholastic performance and academic achievement (Su et al., 2020), often resulting in life-long negative impacts on vocational and social outcomes later in life (Shan et al., 2020; Timmer et al., 2023; Yadav et al., 2023).

Fortunately, research shows that even babies born with HL can develop communication skills on par with their normal hearing peers, provided they receive appropriate intervention before 6 months of age (Ching et al., 2017). For this reason, INHS programmes have been set up in developed countries to ensure that those with HL are identified and receive timeous interventions, giving them access to sound and the opportunity to develop communication skills alongside their normal-hearing peers (Jones et al., 2018; Yoshimura et al., 2024). Unfortunately, in SA, most health facilities do not provide INHS because of the shortage of healthcare resources such as audiologists coupled with the early discharge of newborns before screenings can be conducted (Phanguphangu et al., 2024, 2025). While research has explored the clinical utility of nurse- and community healthcare worker- (CHW) INHS programmes, there is limited evidence regarding their reliability in conducting the screenings. Furthermore, these studies have demonstrated high false-positive or high initial referral rates. While previous research has shown that vernix in the ear canal in the first few days of life contributes to high referral rates (Schwarz et al., 2023), there are limited studies exploring the reliability of nurse- or CHW-administered screening, especially in older babies that may be screened at immunisation clinics when the infants are around 6 weeks of age and the vernix may have dried out in the ear canals (De Kock et al., 2016; Friderichs et al., 2012; Kgare & Joubert, 2024).

Given the limited number of audiologists in the country, the negative impact of untreated childhood HL, and systemic challenges that inhibit the implementation of audiologist-led INHS programmes, SA needs to move from audiologist-led INHS programmes and adopt task-shifting practices where other professionals such as nurses or CHWs champion these programmes with audiologists playing a supportive role where they provide diagnosis and subsequent interventions (Phanguphangu et al., 2024). This is especially important given that SA is at the dawn of its National Health Insurance, which warrants equitable access to healthcare for all. Thus, there is a need to establish the reliability of nurses in conducting INHS. This is especially important because most INHS programmes globally are becoming mainly nurse- or CHW-led initiatives (Health Professions Council of South Africa, 2018; Phanguphangu et al., 2024, 2025). In addition, given the impact of untreated HL during infancy, urgent solutions to facilitate the early hearing detection and intervention (EHDI) of congenital HL in SA are needed to mitigate its impact.

## Research methods and design

### Study aim

This study aimed to determine the reliability of nurse-administered INHS at a primary health care (PHC) clinic.

### Study objectives

This study sought to determine the percentage agreement between the audiologist- and nurse-administered INHS using otoacoustic emissions screening tests.

### Study design

This was a pilot study that employed a repeated-measures within-subject design (Leedy & Ormrod, 2021). This design allowed the researcher to assess the reliability of INHS independently conducted by two nurses on the same participants, facilitating the essential test-retest, and comparisons both between and within participants.

### Study setting

This study was conducted at a PHC clinic in SA, which provides immunisations for babies as per the Extended Programme on Immunisations (EPI) (KwaZulu-Natal Department of Health, 2022). It was conducted before the implementation of a clinical trial aimed at evaluating the feasibility of integrating nurse-administered INHS into the immunisation programme at the clinic (Phanguphangu & Ross, 2025). The coverage rate of all immunisations from birth to 1 year is over 90% (95% confidence interval: 83.4–92.6) in the region where this study was conducted (KwaZulu-Natal Department of Health, 2022). A situational analysis of the clinic showed that it did not provide INHS services previously because of the lack of equipment and limited training of the nurses. The nursing workforce at the clinic includes a facility Nursing Manager (NM) and Registered Nurses (RN). All patients seen at the clinic who require audiological care are referred directly to a local hospital, which offers a full range of audiology services, including specialist paediatric audiology services.

### Study population and sampling strategy

Non-probability purposive sampling was used to recruit and select two RNs to participate in this study (Leedy & Ormrod, 2021). This sampling strategy was used as it allowed the researcher to specifically target a particular subset of the population with certain characteristics and most relevant to their research question. In this case, prospective participants had to be providing immunisations for babies below 12 months for at least 2 years and also hold an RN Certification with the South African Nursing Council (SANC). By selecting the nurses who were already conducting immunisations, the researcher was targeting the group of nurses at the PHC who were already working in the area where the research would be integrated.

In addition, a sample of babies who were scheduled to receive immunisations was also included as participants. An online sample size calculator was used to determine the appropriate sample size required for this study using the following parameters: expected kappa of 0.8, associated with an almost perfect agreement, a 0.05 precision level, 0.5 proportion of outcome, 95% confidence level (Arifin, 2025). Based on these parameters, 500 ears were required for the full study. However, because this was a pilot study, only 20% of the required sample size was recruited, i.e., *n* = 100 ears.

Probability systematic random sampling (Leedy & Ormrod, 2021) was used to select the 50 infants (*n* = 100 ears) scheduled to receive their sixth-week immunisations at the clinic during the study period; every 3^rd^ infant whose parents or legal guardians consented (this was taken as proxy for their baby) to participate in the study was enrolled in the study. To be included in the study, the baby had to be receiving the sixth-week immunisation and having parents or legal guardians who were old enough to give written informed consent. Babies with congenital malformations and those who were critically ill were excluded.

### Intervention

An INHS protocol recommended by the HPCSA was used (Health Professions Council of South Africa, 2018). This protocol included the training of screeners, that is, nursing personnel, and providing them with DPOAE screening equipment. Two nurses who conducted the immunisations underwent a 10-day training, delivered in one-hourly training every Monday for 10 consecutive days. The researcher provided this training, which aimed to improve the nurses’ theoretical and practical skills to conduct INHS using the HPCSA EHDI Programme Hearing Screening Curriculum (Health Professions Council of South Africa, 2018). This training focused on educating these nurses to improve their knowledge of: (1) the principles and rationale of EHDI, (2) anatomy and physiology of the ear, (3) electrophysiological screening measures, (4) ability to conduct a screening according to testing protocols, (5) how to conduct pre- and post-screening counselling, recommendations and making appropriate referrals as per screening protocols, (6) troubleshooting in the event of challenges encountered during screening, (7) promotion and prevention activities related to EHDI, (8) collaboration and consultation with relevant stakeholders in EHDI and (9) administration and record keeping. After undergoing training, these nurses underwent a theoretical examination as per the HPCSA curriculum, and to be certified as competent screeners, each of them had to score at least 80%. Both nurses obtained scores > 80% in this assessment. This theoretical training was followed by practical demonstrations of how to conduct screening using a DPOAE screener on a baby scheduled to receive immunisations. The practical demonstration was led by an audiologist, who screened five babies using a DPOAE screener while they observed. In addition to the observation of the audiologist, the nurses also had an opportunity to practice the screening on the five babies while the audiologist supervised them to ensure patient safety and that they were conducting the screening correctly.

### Data collection tools

The following tools were used during the data collection phase:

- The Biologic AuDx DPOAE screening device along with neonatal ear tips was used to conduct INHS. DPOAE screening was selected for its cost-effectiveness, affordability and the rapid results it provides, typically within 30 s per ear (Martin & Clark, 2014). This efficiency made it particularly suitable for busy immunisation programmes. Furthermore, DPOAE screening requires minimal training, and the equipment is generally compact and portable, allowing for easy transport to immunisation clinics. However, it is important to observe that DPOAEs often result in high false positive rates as they can be affected by environmental noise or middle ear issues (Young & Ng, 2023). In addition, DPOAEs have limited abilities to detect auditory neuropathy spectrum disorder (Bennett et al., 2023), mild or low-frequency loss, and often categorise normal ears as abnormal if there is fluid in the middle ear, even if the cochlea is normal (Bennett et al., 2023). Moreover, DPOAEs are limited in identifying other cases of HL such as those arising from auditory neuropathy spectrum disorder (Bennett et al., 2023). Nevertheless, the Biologic AuDx which operates on a battery that can be recharged using electricity enabling it to run on battery power for extended periods was used in this study. This was especially advantageous given that SA sometimes experiences frequent power outages – the rechargeable battery ensures that screening can proceed uninterrupted during power cuts, as the equipment remains functional when adequately charged. To ensure valid and reliable screening outcomes, the DPOAE screener was calibrated according to manufacturer’s instructions and complied with the international standards.
- A screening register was developed that recorded participants’ demographic characteristics such as date of birth, sex, contact details of the parents and screening outcomes per year.

### Data collection procedures

Prior to commencement of the study, ethics approval was sought and obtained from a university and provincial health research ethics committees. In addition, permissions to conduct the study were obtained from the management of the clinic where data collection took place. The Health Minister’s approval was waived as the study posed minimal risk with undergoing hearing screening and two ethics committees had approved the study.

Once approvals were granted and training was completed, information regarding the hearing screening and its importance was shared with parents or legal guardians who brought their babies for screening each day. Parents or legal guardians who expressed consent to allow their babies to participate in the study were provided with information and signed informed consent as proxy for their babies to be enrolled in the study.

To keep the ambient noise levels down, a room in a separate building within the PHC clinic was identified by the NM and it was designated for immunisations and INHS. In addition, the DPOAE screener features noise level measurement and when the ambient noise is too loud, the screener will inform the screening personnel that there is too much noise in the background to complete the testing. Furthermore, to reduce patient noise and artefacts, nurses started by administering INHS prior to providing immunisations to the babies.

This was a single-stage screening protocol, with a bilateral pass criterion, as follows:

- The first nurse explains the purpose of INHS and the screening procedures to the parents or legal guardians of the baby to be screened. Thereafter, the parents or legal guardians sign an informed consent form for their baby to be screened and to participate in the research study.
- Each nurse independently conducts the hearing screening on each baby and records the results.
- After conducting the INHS, each nurse completes the screening register with demographic information, parent or legal guardian’s contact information (this enabled the researcher to follow up with participants who failed the screening) and screening outcomes.
- After the nurses had independently conducted screening, the researcher (an audiologist with more than 10 years of experience in paediatric audiology) conducted the third DPOAE screening on each baby and recorded the results in the screening register.
- If the participant obtained bilateral ‘Pass’ result, they were discharged and informed to have annual hearing monitoring and reviewing developmental milestones to allow early detection of developmental delays by the audiologist, who screened them after the nurses.
- The screening protocol included screening at four frequencies, that is, 2000, 3000, 4000 and 5000 Hz, and to pass the screening, the patient had to obtain a pass result on ¾ frequencies. These frequencies were selected as screening at higher frequencies (2 kHz – 4 kHz or 2 kHz – 5 kHz) is associated with reduced referral rates, as this frequency range is less influenced by the presence of fluid and debris in the ear canal (Akinpelu et al., 2014).
- If the participant obtained a unilateral or bilateral ‘Refer’ result, the results were recorded in the screening register and the Road to Health Booklet – the audiologist from the local hospital conducted further assessments to determine the aetiology and made the appointments at the referral hospital for further assessment and management. In addition, patients who presented with otitis media were given treatment at the clinic and scheduled for a follow-up appointment within 2 weeks.

### Data management and analysis

To ensure reliability and validity of the results, all the data captured (100%) were independently checked by a nurse and an audiologist, both of whom had more than 5 years of experience verifying research data entries for accuracy and it was confirmed that all the data were accurately captured. After the accuracy of data entries was confirmed, the data were imported to Stata v18 for analysis. Descriptive statistics were used to summarise the data; means of central tendency, that is, mean with standard deviations (SD), were used for continuous variables such as age while percentages and frequency tables were used for categorical data.

In addition to descriptive statistics, Cohen’s kappa statistic was computed to determine the percentage agreement between the audiologist- and nurse-administered screening results. The Kappa value was interpreted as follows: slight (0.00–0.20), fair (0.21–0.40), moderate (0.41–0.60), substantial (0.61–0.80) and almost perfect (0.81–1.00) agreement. This allowed the researcher to determine the reliability of nurse-administered screening in this study and evaluate their ability to correctly identify babies with HL and those with normal hearing.

### Ethical consideration

This study received ethics approval from the University of KwaZulu-Natal’s Biomedical Research Ethics Committee (reference number: BREC/00006710/2024) and the Kwazulu-Natal Department of Health (reference number: KZ_202402_023). It adhered to the ethical principles outlined in the World Medical Association Declaration of Helsinki and all of its later amendments (World Medical Association, 2013). All participants gave written informed consent to participate in the study and for the data to be used for research purposes including publication. The parents or legal guardians of the infants enrolled in this study gave written consent, which served as proxy for their babies, and for the data in this study to be published. Ministerial permissions to conduct the study were not sought given the minimal risk involved with undergoing hearing screening and because two ethics committees approved this study.

## Results

### Participants’ demographic characteristics

An audiologist with more than 10 years of working experience in paediatric audiology and two RNs conducted INHS. The mean age of the three screeners was 34.7 years (s.d.: 2.1 years). In addition, a total of 50 infants (*n* = 100 ears) with a mean age of 6.3 weeks (s.d.: 1.2 weeks) were screened. Forty-six per cent (46%, *n* = 23) of the infants were male while 27 were female and none of the infant participants had any overt risk factors for HL

### Screening outcomes

Thirteen (26%) infants failed the screening of which 11 obtained bilateral refers while 2 were unilateral refers. Four (*n* = 4) of the participants who failed the screening were male and nine (*n* = 9) were female. After the audiologist had confirmed the screening outcomes, all participants who failed the screening were referred to the audiology department at the local hospital for comprehensive audiological assessment. The audiological assessment at the local hospital included an otoscopic examination, middle ear assessment through high-frequency tympanometry, rescreening using DPOAEs, and diagnostic auditory brainstem response or Auditory Steady State Response to ascertain hearing thresholds. Those with conditions that required medical intervention were referred to the paediatric outpatient department at the local hospital. All participants who failed the screening were given appointments and consulted within a week after identification.

Of those who failed the screening, nine babies were identified with otitis media, which was further ascertained by the paediatricians at the local hospital. Among the remaining four babies who failed the initial screening, one baby was diagnosed with a bilateral moderate-to-severe sensorineural HL while the other baby had unilateral severe sensorineural HL. Both babies with HL are currently receiving HL intervention at the hospital. The remaining two babies presented with normal hearing and were discharged from the Audiology department.

Statistical analysis revealed a substantial agreement (*k* = 0.80, *p* < 0.001) between the DPOAE results between the audiologist and Nurse1, and an almost perfect agreement between the Audiologist and Nurse2 (*k* = 0.89, *p* < 0.001). Overall, an almost perfect agreement was found between the audiologist-administered screening and the otoacoustic emissions (OAE) screening results from the two nurses (*k* = 0.81, *p* < 0.001).

Table 1 summarises the interrater reliability results from the study and shows that the results were statistically significant demonstrating high reliability.

**TABLE 1:** Cohen’s Kappa statistic.

Participant	Agreement (%)	Kappa	SE	*p*
Audiologist	Ref	Ref	Ref	Ref
Nurse 1	93	0.80	0.09	< 0.001
Nurse 2	96	0.89	0.10	< 0.001
Overall	-	0.81	-	< 0.001

SE, standard error; Ref, reference variable.

## Discussion

This is the first study to determine the reliability of nurse-administered INHS at primary healthcare in SA. Findings from this study showed a high initial referral rate (26%) and an almost perfect agreement (*k* = 0.81, *p* < 0.001) between the audiologist- and nurse-administered screening, demonstrating high reliability of nurse-administered INHS using DPOAE screening tests. The initial referral rate (26%) obtained in this study is higher than the 7–9.5% reported in previous studies in similar settings in SA (De Kock et al., 2016; Friderichs et al., 2012; Kgare & Joubert, 2024) and the national guidelines of < 5% initial referral rate (Health Professions Council of South Africa, 2018). While the high referral rate reported in the previous study was attributed to the presence of vernix in the external ear canals as their participants were younger, the participants in this study were older and mainly failed the INHS screening because of the presence of middle ear infections. Previous research supports the notion that middle ear infections can affect DPOAE testing, as the presence of fluid in the middle ear cavity affects the recording of acoustic emissions in the ear canals of patients with otitis media (Martin & Clark, 2014).

While a previous study has recommended the use of both DPOAEs and AABR to reduce the initial high referral rates (De Kock et al., 2016), in reality, AABR technologies are more expensive to procure and require expensive consumables to operate (Phanguphangu & Ross, 2021). In addition, unlike DPOAEs, which can be obtained within 30 s per ear in infants who are calm and quiet, AABR screening takes longer to complete (Akinpelu et al., 2014; Ngui et al., 2019; Sheng et al., 2021) and often requires the infant to be very still with minimal movement to reduce artefacts that may interfere with testing (Esaki et al., 2024). This is not ideal in a busy immunisation clinic where infants can be very active and thus makes it challenging to conduct AABR screening. As a result, the use of AABR as the main screening technologies can deter nurses from adopting their use (Phanguphangu et al., 2025).

Our findings demonstrate high reliability of nurse-administered INHS using DPOAEs, highlighting the diverse role of the PHC nurse. In addition, these findings demonstrate that when adequately trained, nurses can reliably screen and identify infants with congenital or early-onset HL using DPOAE screening tests. Given the high reliability obtained in our study, our findings are useful in the successful planning of secondary prevention programmes for congenital HL, such as the early identification and management of this HL. Currently, there is limited provision of INHS in both hospitals and PHC clinics in SA (Louw et al., 2024; Phanguphangu et al., 2024, 2025), mainly because of systemic challenges such as the early discharge of newborns before INHS can be provided by audiologists. For instance, a recent study found that a significant number of babies born over the weekend, or those born overnight and discharged early in the morning did not receive screening as the audiologists were not at work to screen them (Phanguphangu et al., 2024). In addition to the early discharge of newborns, some hospitals only perform risk-based screenings, where newborns are only screened if they meet the risk-based criteria, that is, if they have known risk factors for HL (Phanguphangu et al., 2024). This is despite research evidence showing that over a third of congenital HL occurs with no identifiable risk factor (Al-Ani, 2023). Thus, providing risk-based INHS only could miss identifying over a third of babies with congenital HL.

Despite research highlighting the scarcity of audiologists in SA (Pillay et al., 2020) and studies in other countries showing that nurses and other mid-level workers such as CHWs can be trained to provide INHS (Russ et al., 2010; Yoshimura et al., 2024), there is still limited implementation trials exploring the reliability of nurse-administered INHS, with most implementation studies focusing on audiologist-administered screening across different levels of care (Phanguphangu et al., 2025). The SA needs to transition from audiologist-led INHS to nurse- or CHW-led programmes. By adopting this shift, the already limited audiologists will have more time to focus on providing diagnostic assessments and intervention rather than screening which can be performed at lower levels of care (Phanguphangu et al., 2025). In addition, by using nurses as the primary drivers of INHS programmes, healthcare facilities may finally realise the programmatic implementation of INHS while also reducing their expenditure associated with additional staff employment as the nurses are already employed at these facilities (Phanguphangu & Ross, 2021; Phanguphangu et al., 2024). Furthermore, using nurses as the primary drivers in both hospital and community-based settings has the potential to achieve higher coverage of INHS and to lower the identification of congenital HL in line with the recommended local best-practice guidelines (Phanguphangu et al., 2024, 2025).

Findings from this study are important for the future development, implementation and management of congenital HL, including the inclusion of nurses and CHWs as important team members in its identification and management. However, it is important to note that previous research has highlighted the resistance from nurses, who complain about being overburdened with work and viewing INHS as something out of their scope of practice, consequently leading to their resistance towards its integration in their daily practices (Khan et al., 2018; Mohamed et al., 2022; Naidoo & Khan, 2022; Sharma et al., 2022). Thus, future research should focus on raising awareness of the diverse role of nurses and the valuable contribution they can potentially make in the early identification and management of congenital HL. The use of implementation science and change theory in future research, which includes the training and enhancing stakeholder buy-in from nurses to incorporate INHS in their daily practices are further important factors to consider in future implementation programmes. Furthermore, the incorporation of INHS into the job descriptions of nurses working in immunisation programmes should be considered in future research, as this could mandate nurses to incorporate INHS into their daily practices and mitigate their resistance to providing formalised INHS in the future.

In addition to the inclusion of INHS in the job descriptions, the Road to Health Booklet also requires a revision to specifically include formalised and instrumental INHS, using DPOAEs or AABR, which should be conducted during scheduled immunisation visits. Currently, the Road to Health booklet includes hearing and/or communication under developmental screening. However, this screening is not formalised using screening equipment but rather subjective questions that are directed to the primary caregivers of babies, which may lead to HL being missed. The incorporation of INHS into job descriptions and the revisions of the Road to Health booklet are specifically important given that the Package of Services at PHC level already includes the provision of hearing screening by nurses, and the SANC training curriculum also stipulates that nurses should be trained on how to conduct screening for HL among infants and young children (Dookie & Singh, 2012; South African Nursing Council, 1978).

By including INHS in their job descriptions, nurses will then be mandated to provide this much needed service. However, it is important to note that this is a complex health systems challenge that goes beyond the scope of the the article and needs more research to carefully address. Thus, future research should focus on conducting workload assessments to determine where resources can be reallocated or streamlined to include INHS, followed by training and support of the nurses on how to conduct INHS, feasibility studies to determine the implementation process, empowerment and recognition of nurses to take ownership of INHS, collaborative practice with audiologists to provide technical support and enhance implementation success, and impact evaluations to assess the implementation process and future sustainability of these nurse-administered programmes.

### Study’s limitations

This study has limitations to consider. Firstly, it being a pilot study that was conducted at a single facility with purposely selected nurses limits the generalisability of the findings to all nurses and other settings. Secondly, the smaller sample size of infants screened in this study could also over- or underestimate the reliability of nurse-administered screening obtained in this study. Nevertheless, this study used robust statistical tests, that is, Cohen’s *kappa* to calculate the percentage agreement between the audiologist- and nurse-administered INHS, which increases the strength of our findings.

### Recommendations

Based on these findings, the following recommendations were made:

- Future implementation studies should explore the feasibility of implementing nurse-driven INHS programmes and integrating INHS into immunisation clinics within PHC clinics and other contexts such as maternity wards, nursery, and neonatal intensive care units.
- A collaborative, task-shifting approach should be adopted that includes the capacitation of PHC nurses and the provision of formalised screening equipment, coupled with the support by audiologists to ensure the long-term sustainability of nurse-administered INHS.
- Future studies should explore the incorporation of INHS into the job descriptions of PHC nurses responsible for providing immunisations as this will ensure a systematic and programmatic integration of INHS into pre-existing clinical services at PHC levels, and further legally oblige the nurses to provide the INHS at this level.

## Conclusion

Our findings demonstrate that nurses can provide reliable INHS, and timing the provision of INHS with the scheduled immunisations may offer a better opportunity to ensure the early detection of congenital HL within the local guidelines of identification by 6 weeks of age.
