# Addendum: Reliability of nurse-administered infant hearing screening using otoacoustic emissions

**DOI:** 10.4102/sajcd.v73i1.1177

**Published:** 2026-04-15

**Authors:** Mukovhe Phanguphangu, Andrew J. Ross

**Affiliations:** 1 Department of Family Medicine, College of Health Sciences, University of KwaZulu-Natal, Durban, South Africa

In the published article Phanguphangu, M., & Ross, A.J. (2025). Reliability of nurse-administered infant hearing screening using otoacoustic emissions. *South African Journal of Communication Disorders, 72*(1), a1092. https://doi.org/10.4102/sajcd.v72i1.1092, three updates were required regarding the (1) updated referral rate (26% vs. 23%), (2) updated unit of analysis (infants vs. ears) and (3) updated statistical reporting.

## Updated referral rate (26% vs. 23%)

The corrected referral rate uses the appropriate denominator (number of ears screened). This adjustment does not change the interpretation of screening outcomes or the reliability estimates. The study’s discussion and conclusions remain supported, as the primary objective was to evaluate inter-tester reliability, not screening yield.

## Updated unit of analysis (infants vs. ears)

Terminology in the published version alternates between ‘infants’, ‘babies’ and ‘ears’. The consistent unit of analysis for this study is ears, as inter-rater reliability (Kappa statistics) was conducted at the ear level. The following sections should therefore use the term ‘ears’ for clarity and consistency:

Sections requiring correction:

1. *Research methods and design (study design and statistical analysis)*: Any reference to reliability being calculated per ‘infant’ should be amended to specify that agreement was calculated per ‘ear’.
2. *Results (reliability outcomes)*: All agreement, referral and screening outcomes should refer to ‘ears screened’ rather than ‘infants screened’.
3. *Table 1:* The denominator and all outcome descriptors should reflect ears as the analytic unit.

**TABLE 1:** Cohen’s Kappa statistic.

Participant	Agreement (%)	Kappa	95% CI	SE	*p*-value
Audiologist	Ref	Ref	Ref	Ref	Ref
Nurse 1	93	0.80	0.62–0.98	0.090	< 0.001
Nurse 2	96	0.89	0.69–1.00	0.100	< 0.001
Overall	-	0.81	0.64–0.98	0.087	< 0.001

SE, standard error; Ref, reference variable; CI, confidence interval.

Sections not requiring substantive revision:

1. Background and rationale sections that refer generally to ‘infants‘ as the target population may remain unchanged, as these describe the population screened rather than the analytic unit.
2. The discussion and conclusions remain valid because the reliability estimates were always calculated correctly at the ear level; the issue was terminological clarification rather than analytic error.

## Updated statistical reporting

Adding 95% confidence intervals (CI) and *p*-values enhances transparency and aligns the article with best-practice statistical conventions. The magnitude and interpretation of agreement remain unchanged: Audiologist vs Nurse 1, *k* = 0.80 (95% CI: 0.62–0.98; *p* < 0.001); Audiologist vs Nurse 2, *k* = 0.89 (95% CI: 0.69–1.00; *p* < 0.001); overall agreement, *k* = 0.81 (95% CI: 0.64–0.98; *p* < 0.001). These values continue to indicate substantial to near-perfect agreement and do not alter the study’s conclusions.

Table 1 has been updated to include corrected and additional data. These changes align with statistical reporting guidelines, which recommend presenting the Kappa statistic (Cohen’s Kappa) with the 95% CI to accurately reflect the distribution of the effect estimate.

## Overall scientific integrity

The statistical analyses were conducted correctly at the ear level in the original submission. The addendum, therefore, clarifies terminology to align the written description with the analysis. Thus, none of the updates:

- changes the direction or magnitude of the reliability findings
- alters the interpretation of agreement levels
- modifies the study’s conclusions
- affects the validity of the recommendation that nurse-administered otoacoustic emissions screening demonstrates strong reliability when appropriately trained and supervised.
